# Factors Associated with Treatment and Survival of Early Stage Pancreatic Cancer in the Era of Modern Chemotherapy: An Analysis of the National Cancer Database

**DOI:** 10.1089/pancan.2020.0011

**Published:** 2020-09-21

**Authors:** Michael D. Watson, Jennifer L. Miller-Ocuin, Michael R. Driedger, Michael J. Beckman, Iain H. McKillop, Erin H. Baker, John B. Martinie, Dionisios Vrochides, David A. Iannitti, Lee M. Ocuin

**Affiliations:** ^1^ Division of Hepatobiliary and Pancreatic Surgery, Department of Surgery, Atrium Health, Charlotte, North Carolina, USA.; ^2^ Department of Surgery, Wake Forest University School of Medicine, Winston-Salem, North Carolina, USA.; ^3^ Division of Hepatobiliary and Pancreas Surgery, Department of Surgery, Mayo Clinic, Rochester, Minnesota, USA.; ^4^ Department of Surgery, The Johns Hopkins Medical Institutions, Baltimore, Maryland, USA.

**Keywords:** pancreatic cancer, early clinical stage, treatment, nonoperative, surgery

## Abstract

**Background:** Underutilization of operative management of early stage pancreatic cancer is associated with sociodemographic variables, including age, race, facility type, insurance, and education. It is currently unclear how these variables are associated with survival in patients who undergo surgery.

**Methods:** Patients with clinical stage I pancreatic adenocarcinoma were identified within the National Cancer Database (2010–2016). Utilization of surgery and nonoperative management was determined. Nonclinical factors associated with nonoperative management were identified by multivariable analysis. The association between nonclinical factors and survival was assessed in patients who received operative management.

**Results:** A total of 17,833 patients with clinical stage I pancreatic cancer were identified, and 41.2% underwent operative intervention. Approximately 46% of nonoperatively managed patients lacked a contraindication. Operatively managed patients had longer overall survival (OS) than those who were nonoperatively managed or untreated (25.1 months vs. 11.1 months vs. 5.1 months, *p* < 0.0001). Factors associated with nonoperative management included age, black/Hispanic race, nonacademic facilities, nonprivate health insurance, lower education level, and lower income. In operatively managed patients, nonclinical factors associated with lower OS included Medicaid (hazard ratio [HR] 1.27) and treatment at nonacademic facilities (HR 1.20–1.22). Patients on Medicaid received less adjuvant therapy and had higher 30- and 90-day mortality rates. Patients treated at nonacademic facilities received less neoadjuvant therapy, had worse pathologic outcomes, and had higher 30- and 90-day mortality rates.

**Conclusions:** Surgical management is underutilized in clinical stage I pancreatic cancer. Primary insurance payor and facility type appear to be associated with OS in patients who undergo operative management.

## Synopsis

We identified patients with early stage pancreatic cancer by using the National Cancer Database (2010–2016), and we report that 41% of patients underwent surgery with improved overall survival compared with those who received nonoperative management. Sociodemographic factors were predictive of nonoperative management but not survival in patients who underwent surgery, with the exception of Medicaid and care at nonacademic facilities.

## Introduction

Pancreatic adenocarcinoma (PDAC) is the 11th most common malignancy diagnosed in the United States but it represents the 3rd most common cause of cancer death, with more than 57,000 cases and 47,000 deaths estimated in 2020.^1^ Despite advancements in multimodality care, prognosis remains poor, and the overall 5-year survival is only 9%.^1^

Pancreatectomy is an essential component in the management of PDAC and must be incorporated into treatment with curative intent.^2–4^ Over the past several decades, mortality after pancreatectomy has decreased to <2% in high-volume centers.^5^ In 2007, Bilimoria et al. reported on the underutilization of pancreatectomy in patients with early stage PDAC.^3^ In their analysis of the National Cancer Database (NCDB), the authors report that 70% of patients with clinical stage I disease did not undergo pancreatectomy. The majority of patients had no documented contraindications to surgery and were simply “not offered surgery.” The authors identified several sociodemographic factors that were associated with nonoperative management, including age, nonprivate health insurance, lower income, and lower level of education. This has been confirmed in several other studies using older data from within the Surveillance, Epidemiology, and End Results (SEER) database (2004–2011),^2^ as well as the NCDB (2003–2012),^6^ NCDB 2004–2015,^7^ SEER 1992–2002,^8^ and institutional data (2004–2013) from a high-volume tertiary center.^9^ The management of pancreatic cancer has evolved over the past several years, with incorporation of neoadjuvant therapy,^4,10–12^ prehabilitation,^13^ and minimally invasive approaches,^14–18^ and these data may not be applicable to the modern era.

The primary objective of this study was to re-evaluate the NCDB within the era of modern multimodality management of early stage PDAC for utilization of operative and nonoperative treatment strategies and the impact of these strategies on survival. Our secondary objectives were to identify variables associated with nonoperative management and to evaluate the effect of these variables on survival in patients who underwent surgery. No studies to our knowledge have addressed these questions specifically in the current era of PDAC treatment.

## Methods

### Institutional assurances

Our Institutional Review Board has deemed that retrospective analyses of public, anonymized datasets are exempt from review.

### Patient identification and selection

The NCDB was queried for patients with adenocarcinoma of the pancreas diagnosed between 2010 and 2016. The NCDB is a joint project of the American Cancer Society and the Commission on Cancer (CoC) of the American College of Surgeons that includes more than 1500 cancer programs in the United States and Puerto Rico. Approximately 70% of newly diagnosed cancer cases in the United States are reported to NCDB. Patients were identified by using the *International Classification of Diseases for Oncology* (ICD-O) codes 25.0, 25.1, 25.2, 25.3, 25.4, 25.7, 25.8, and 25.9), histology codes consistent with adenocarcinoma, adults (age ≥18), and tumors classified as clinical stage I (T1/T2N0M0) by the American Joint Committee on Cancer (AJCC, seventh edition).

### Variables of interest

Data abstracted included demographics (age, sex, race, facility type, primary insurance payor, education level quartile [as determined by the 2016 United States Census data]), median income quartile (as determined by the 2016 United States Census data), population density (metro, urban, or rural based on the United States Department of Agriculture Economic Research Service definition), treating facility type, comorbidities, tumor characteristics (clinical T classification, tumor location, serum carbohydrate antigen 19-9 [CA19-9] level), details of treatment (receipt and timing of chemotherapy and/or radiotherapy and/or hormone therapy and/or immunotherapy and/or type of surgery), histopathology (pathologic T, pathologic N, nodal yield, lymph node ratio, margin status), and postoperative outcomes (30-/90-day mortality, 30-day readmission). A patient was identified as undergoing pancreatectomy based on site-specific coding in the database. Reasons for nonoperative management included “surgery not offered,” “not recommended due to comorbidities,” “patient refusal,” “death prior to surgery,” and “unknown reason.”

### Statistical analysis

Continuous variables were compared with two-tailed Student's *t*-test, and categorical variables were compared with the chi-squared test. Overall survival (OS) was defined as time between diagnosis and either death or last follow-up. OS was estimated with the Kaplan-Meier method, and groups were compared with the log-rank test. For *post hoc*, pairwise comparisons of survival between three groups, the Benjamini-Hochberg method was used.^19^ A multivariable logistic regression was used to determine factors associated with nonoperative management with age, sex, race, facility type, primary insurance payor, population density of home zip code, distance from the hospital, education level of home zip code, median income of home zip code, Charlson-Deyo score, clinical T classification, tumor primary site, and CA19-9 level used as independent variables. A Cox proportional hazards model was used to assess the association of the previously mentioned variables with survival in both univariate and multivariable fashion. Statistical significance was defined as α < 0.05. All statistical analysis and figure creation was performed with R software (version 3.6.1; The R Foundation, Vienna, Austria).

## Results

### Patient demographics

Overall, 162,877 patients in the NCDB were diagnosed with PDAC between 2010 and 2016, of whom 17,833 (10.9%) had clinical stage I disease. The median age at diagnosis was 72 years, 51.7% were female, and 80.7% were white (Table 1). Forty-four percent of patients were treated at academic/research facilities, and 25.8% had private health insurance. Most tumors were located in the pancreatic head (70.4%).

**Table 1.** Baseline Demographics and Clinical Features of Patients With Early Stage Pancreatic Cancer

Variable	n (%)
Total patients	17,833
Age, years	
Mean ± SD	71.2 ± 11.4
Median (IQR)	72 (63–80)
<55	1480 (8.3)
56–65	3947 (22.1)
66–75	5425 (30.4)
76–85	5108 (28.6)
>85	1873 (10.5)
Sex	
Female	9220 (51.7)
Male	8613 (48.3)
Race	
White	14396 (80.7)
Black	1886 (10.6)
Hispanic	803 (4.5)
Asian	344 (1.9)
Other/Unknown	404 (2.3)
Facility type	
Academic/Research	7853 (44.0)
Community	7504 (42.1)
Integrated network	2398 (13.4)
Other/Unknown	78 (0.4)
Insurance	
Private	4602 (25.8)
Medicare	11597 (65.0)
Medicaid	751 (4.2)
Government	262 (1.5)
None	342 (1.9)
Unknown	279 (1.6)
No HSD in zip code	
<6.3%	4437 (24.9)
6.3–10.8%	5072 (28.4)
10.9–17.5%	4663 (26.1)
>17.6%	3460 (19.4)
Missing	201 (1.1)
Median income	
> $63,333	6096 (34.2)
$50,354 to $63,332	4148 (23.3)
$40,227 to $50,353	4127 (23.1)
< $40,227	3231 (18.1)
Missing	231 (1.3)
Charlson-Deyo score	
0	11142 (62.5)
1	4654 (26.1)
2	1326 (7.4)
≥3	711 (4.0)
Clinical T	
T1	4429 (24.8)
T2	13404 (75.2)
Primary site	
Body/Tail	3151 (17.7)
Head	12554 (70.4)
Not specified	2128 (11.9)
CA19-9	
<38 U/mL	3338 (18.7)
≥38 U/mL	7117 (39.9)
Missing	7378 (41.4)

CA19-9, carbohydrate antigen 19-9; HSD, high school diploma; IQR, interquartile range; SD, standard deviation.

### Treatment of early stage PDAC

**FIG. 1.**
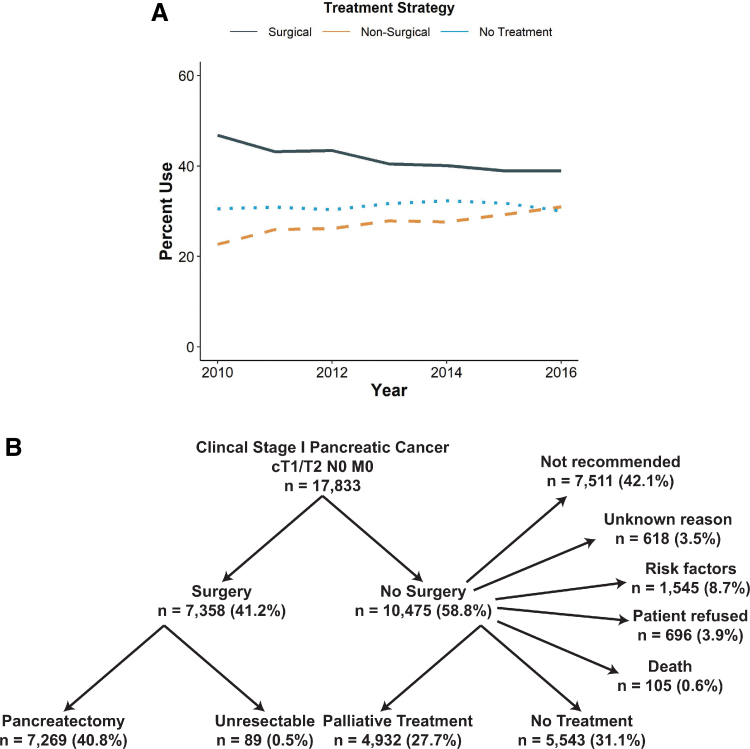
Treatment strategy for patients with early stage pancreatic cancer over the study period (2010–2016). **(A)** Trends over time (black line: surgery; orange line: nonsurgical, chemotherapy; blue line: no treatment). **(B)** Distribution of management categories and documented rationale for treatment decisions.

Treatment trends over the study period indicate that the rate of surgical treatment decreased from 46.8% to 38.9% (black line), whereas the rate of nonoperative management increased from 22.6% to 30.9% (orange line). The rate of patients receiving no treatment remained unchanged (∼30%, blue line; Fig. 1A).

Of the 17,833 patients identified with early stage PDAC, 41.2% were offered surgery, the majority of who underwent successful pancreatectomy (Fig. 1B). Of the 10,475 patients who did not undergo surgery, 8.7% were excluded for comorbidities, 3.9% refused, and 0.6% died. The remaining patients were either not recommended for surgery (*n* = 7511, 42.1%) or had no documented reason (*n* = 618, 3.5%), and 5543 (31.1%) of these patients received no treatment. Of the 27.7% patients who received palliative treatment, the majority received chemotherapy (*n* = 4309). In addition, 2332 patients received radiotherapy, 17 received hormonal therapy, and 23 received immunotherapy.

### Impact of treatment strategy on survival

**FIG. 2.**
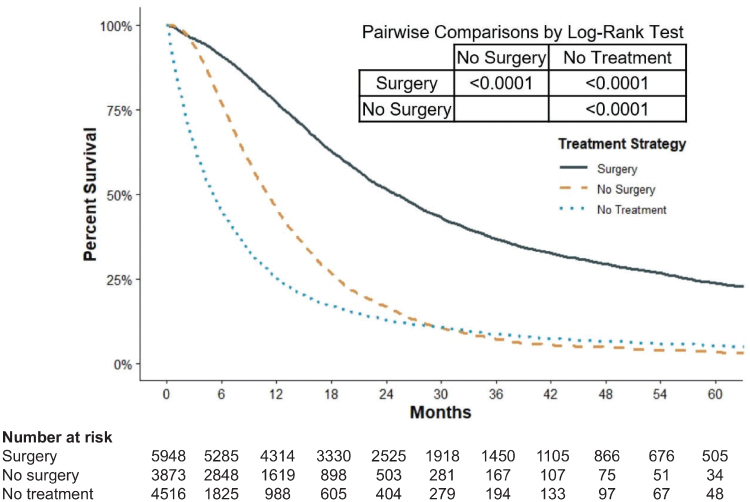
Kaplan-Meier estimates for overall survival of patients with early stage pancreatic cancer who underwent surgery, received nonsurgical management, or received no treatment.

Evaluation of the association between treatment strategy and patient OS demonstrated a median follow-up of 11.4 months. Patients who underwent surgical management had longer median OS (25.1 months) than those who underwent nonoperative management (11.1 months) or received no treatment (5.1 months; all *p* < 0.0001; Fig. 2). One- and 5-year survival was improved in patients who received surgical management (76.9% [1-year]/24% [5-year]) compared with those who received nonsurgical intervention (45.8% [1-year]/3.5% [5-year]) or no treatment (25.4% [1-year]/5.5% [5-year]). The adjusted hazard ratio (HR) for nonoperative management was 2.12 (95% CI 2.02–2.23), and the HR for no treatment was 3.25 (95% CI 3.09–3.41, all *p* < 0.0001).

### Factors predicting operative versus nonoperative management

After excluding patients who refused surgery, died, or had risk factors precluding operative intervention, we identified patient variables that were associated with nonoperative management. Univariate factors included increasing age, black or Hispanic race, care at community facilities, nonprivate health insurance, urban population density, lower education level, median income < $63,333, Charlson-Deyo score ≥3, cT2 tumors, pancreatic head tumors, and CA19-9 levels ≥38 U/mL (Table 2).

**Table 2.** Univariate and Multivariable Analysis of Factors Associated with Nonoperative Management of Early Stage Pancreatic Adenocarcinoma

Variable	Univariate			Multivariable		
	OR	95% CI	p	OR	95% CI	p
Age, mean ± SD	1.05	1.05–1.06	**<0.001**	1.07	1.06–1.07	**<0.001**
Sex						
Female	Ref.	Ref.	Ref.	Ref	Ref.	Ref.
Male	0.93	0.88–1.00	**0.037**	1.06	0.99–1.13	0.125
Race						
White	Ref.	Ref.	Ref.	Ref.	Ref.	Ref.
Black	1.26	1.14–1.40	**<0.001**	1.51	1.34–1.70	**<0.001**
Hispanic	1.26	1.08–1.47	**0.003**	1.28	1.08–1.51	**0.005**
Asian	0.89	0.70–1.11	0.297	0.91	0.71–1.17	0.474
Other/Unknown	1.22	0.99–1.51	0.059	1.35	1.08–1.70	**0.010**
Facility type						
Academic/Research	Ref.	Ref.	Ref.	Ref.	Ref.	Ref.
Community	1.45	1.36–1.56	**<0.001**	1.39	1.29–1.50	**<0.001**
Integrated network	1.04	0.94–1.14	0.479	0.98	0.88–1.09	0.759
Unknown	0.41	0.24–0.67	**0.001**	3.12	1.78–5.31	**<0.001**
Insurance						
Private	Ref.	Ref.	Ref.	Ref.	Ref.	Ref.
Medicare	1.87	1.74–2.02	**<0.001**	0.84	0.76–0.92	**<0.001**
Medicaid	1.49	1.27–1.76	**<0.001**	1.58	1.27–1.76	**<0.001**
Government	2.01	1.54–2.63	**<0.001**	1.36	1.02–1.83	**0.036**
None	1.66	1.32–2.10	**<0.001**	1.86	1.44–2.40	**<0.001**
Unknown	3.20	2.43–4.25	**<0.001**	1.90	1.41–2.57	**<0.001**
Population density						
Metro	Ref.	Ref.	Ref.	Ref.	Ref.	Ref.
Urban	1.14	1.04–1.24	**0.004**	1.05	0.95–1.16	0.345
Rural	0.98	0.78–1.22	0.832	0.86	0.67–1.10	0.227
Distance, mi ± SD	1.00	1.00–1.00	**0.047**	1.00	1.00–1.00	0.540
No HSD in zip code						
<6.3%	Ref.	Ref.	Ref.	Ref.	Ref.	Ref.
6.3–10.8%	1.07	0.98–1.17	0.13	1.02	0.93–1.13	0.644
10.9–17.5%	1.21	1.11–1.32	**<0.001**	1.11	0.99–1.25	0.076
>17.6%	1.40	1.27–1.54	**<0.001**	1.22	1.07–1.41	**0.004**
Missing	1.05	0.77–1.43	0.761	0.58	0.22–1.45	0.256
Median income						
> $63,333	Ref.	Ref.	Ref.	Ref.	Ref.	Ref.
$50,354 to $63,332	1.17	1.07–1.27	**<0.001**	1.12	1.01–1.24	**0.029**
$40,227 to $50,353	1.30	1.20–1.42	**<0.001**	1.19	1.06–1.33	**0.004**
< $40,227	1.46	1.33–1.60	**<0.001**	1.30	1.14–1.50	**<0.001**
Missing	1.17	0.88–1.56	0.272	2.30	1.00–5.66	0.058
Charlson-Deyo score						
0	Ref.	Ref.	Ref.	Ref.	Ref.	Ref.
1	0.90	0.83–0.96	**0.003**	0.84	0.77–0.90	**<0.001**
2	1.00	0.89–1.14	0.953	0.89	0.77–1.02	0.087
≥3	1.22	1.03–1.46	**0.023**	1.16	0.96–1.40	0.130
Clinical T						
T1	Ref.	Ref.	Ref.	Ref.	Ref.	Ref.
T2	1.78	1.66–1.92	**<0.001**	1.81	1.67–1.97	**<0.001**
Primary site						
Body/Tail	Ref.	Ref.	Ref.	Ref.	Ref.	Ref.
Head	1.38	1.27–1.50	**<0.001**	1.40	1.28–1.53	**<0.001**
Not specified	1.88	1.67–2.12	**<0.001**	1.97	1.74–2.24	**<0.001**
CA19-9						
<38 U/mL	Ref.	Ref.	Ref.	Ref.	Ref.	Ref.
≥38 U/mL	1.70	1.56–1.86	**<0.001**	1.60	1.45–1.76	**<0.001**
Missing	1.96	1.80–2.15	**<0.001**	1.82	1.66–2.01	**<0.001**

Bold numbers are for statistical significance (*p* < 0.05).

CI, confidence interval; Distance, distance from hospital; mi, miles; OR, odds ratio; population density, population density where patient lives.

On multivariable analysis, factors that were independently associated with nonoperative management included increasing age, black or Hispanic race, care at community facilities, nonprivate health insurance, lower education level, median income < $63,333, cT2 tumors, pancreatic head tumors, and CA19-9 levels ≥38 U/mL (Table 2).

### Impact of sociodemographic factors on survival in operatively managed patients with early stage PDAC

An unadjusted Cox proportional hazard model demonstrated that age, care at nonacademic facilities, insurance through Medicare or Medicaid, lack of high school graduation of >10.9%, median household income of < $50,353, Charlson-Deyo score ≥1, cT2 tumors, pancreatic head tumors, and serum CA19-9 level of ≥38 U/mL were associated with lower OS (Table 3). After adjusting for demographic and clinical factors, independently associated predictors of worse OS included care at community facility (HR 1.22) or integrated network (HR 1.20), Medicare (HR 1.10) or Medicaid insurance (HR 1.27), median income $40,227 to $50,353 (HR 1.16), Charlson-Deyo score ≥2 (HR 1.28), cT2 tumors (HR 1.17), pancreatic head tumors (HR 1.21), and CA19-9 ≥ 38 U/mL (HR 1.36).

**Table 3.** Unadjusted and Adjusted Cox Proportional Hazards Model Predictors of Survival in Patients Undergoing Pancreatectomy for Early Stage Pancreatic Adenocarcinoma

Variable	Unadjusted			Adjusted		
	HR	95% CI	p	HR	95% CI	p
Age	1.02	1.01–1.02	**<0.001**	1.02	1.01–1.02	**<0.001**
Sex						
Female	Ref.	Ref.	Ref.	Ref.	Ref.	Ref.
Male	1.01	0.95–1.08	0.755	1.03	0.97–1.10	0.320
Race						
White	Ref.	Ref.	Ref.	Ref.	Ref.	Ref.
Black	0.98	0.88–1.09	0.706	0.98	0.87–1.10	0.743
Hispanic	0.92	0.78–1.09	0.340	0.93	0.78–1.10	0.390
Asian	0.77	0.61–0.98	**0.033**	0.74	0.58–0.94	**0.014**
Other/Unknown	0.95	0.76–1.19	0.648	1.04	0.83–1.31	0.735
Facility type						
Academic/Research	Ref.	Ref.	Ref.	Ref.	Ref.	Ref.
Community	1.23	1.15–1.32	**<0.001**	1.22	1.13–1.31	**<0.001**
Integrated network	1.24	1.13–1.36	**<0.001**	1.20	1.09–1.32	**<0.001**
Unknown	0.41	0.25–0.69	**<0.007**	0.76	0.45–1.28	0.297
Insurance						
Private	Ref.	Ref.	Ref.	Ref.	Ref.	Ref.
Medicare	1.35	1.26–1.45	**<0.001**	1.10	1.00–1.20	**0.046**
Medicaid	1.22	1.03–1.43	**0.018**	1.27	1.07–1.50	**0.005**
Government	1.23	0.92–1.63	0.156	1.12	0.84–1.49	0.447
None	1.03	0.82–1.31	0.778	1.08	0.85–1.37	0.547
Unknown	1.33	0.96–1.83	0.087	1.21	0.88–1.68	0.246
Population density						
Metro	Ref.	Ref.	Ref.	Ref.	Ref.	Ref.
Urban	1.05	0.96–1.15	0.295	0.96	0.87–1.06	0.396
Rural	1.17	0.94–1.47	0.167	1.11	0.88–1.14	0.366
Distance (mi ± SD)	1.00	1.00–1.00	0.335	1.00	1.00–1.00	0.947
No HSD in zip code						
<6.3%	Ref.	Ref.	Ref.	Ref.	Ref.	Ref.
6.3–10.8%	1.02	0.94–1.11	0.603	0.99	0.9–1.09	0.876
10.9–17.5%	1.17	1.07–1.27	**<0.001**	1.11	0.99–1.23	0.069
>17.6%	1.14	1.03–1.25	**0.010**	1.08	0.94–1.23	0.272
Missing	1.13	0.84–1.51	0.434	2.97	0.71–12.38	0.135
Median income						
> $63,333	Ref.	Ref.	Ref.	Ref.	Ref.	Ref.
$50,354 to $63,332	1.05	0.96–1.14	0.276	1.00	0.91–1.10	0.972
$40,227 to $50,353	1.22	1.12–1.33	**<0.001**	1.16	1.05–1.30	**0.005**
< $40,227	1.20	1.09–1.31	**<0.001**	1.14	1.00–1.30	0.051
Missing	1.04	0.78–1.38	0.815	0.40	0.10–1.60	0.197
Charlson-Deyo score						
0	Ref.	Ref.	Ref.	Ref.	Ref.	Ref.
1	1.10	1.02–1.18	**0.009**	1.07	1.00–1.15	0.064
2	1.33	1.18–1.50	**<0.001**	1.28	1.13–1.44	**<0.001**
≥3	1.36	1.12–1.66	**0.002**	1.33	1.09–1.63	**0.005**
Clinical T						
T1	Ref.	Ref.	Ref.	Ref.	Ref.	Ref.
T2	1.2	1.13–1.30	**<0.001**	1.17	1.09–1.26	**<0.001**
Primary site						
Body/Tail	Ref.	Ref.	Ref.	Ref.	Ref.	Ref.
Head	1.22	1.12–1.32	**<0.001**	1.21	1.11–1.32	**<0.001**
Not specified	1.02	0.91–1.16	0.705	1.04	0.92–1.18	0.521
CA19-9						
<38 U/mL	Ref.	Ref.	Ref.	Ref.	Ref.	Ref.
≥38 U/mL	1.44	1.32–1.56	<0.001	1.36	1.24–1.48	<0.001
Missing	1.15	1.05–1.25	0.002	1.11	1.02–1.21	0.016

Bold numbers are for statistical significance (*p* < 0.05).

HR, hazard ratio.

### Differences in perioperative and pathologic outcomes of patients with Medicaid or patients treated at nonacademic facilities

Given the association between worse OS after operative intervention in patients with Medicaid insurance or those treated at nonacademic facilities, we compared perioperative and pathologic outcomes between these patient subgroups (Table 4). Compared with patients with private insurance, patients with Medicaid received less adjuvant therapy and had higher 30- and 90-day postoperative mortality. Compared with patients treated at academic/research facilities, those treated at community hospitals or integrated networks received less neoadjuvant therapy and had more frequent nodal involvement, higher lymph node ratios, higher R1 resection rates, longer length of stay, and higher 30- and 90-day mortality rates.

**Table 4.** Differences in Perioperative and Pathologic Outcomes in Patients with Medicaid or Those Treated at Nonacademic Facilities

Variable	Insurance type			Facility type		
	Private (n* = 2507), *n (%)	Medicaid (n* = 329), *n (%)	p	Academic/Research (n* = 3540), *n (%)	Community/Integrated (n* = 3764), *n (%)	p
NAD chemotherapy	445 (17.8)	56 (17.0)	0.744	613 (17.3)	512 (13.6)	**<0.001**
NAD RT	187 (7.5)	25 (7.6)	0.928	268 (7.6)	225 (6.0)	**0.007**
AD chemotherapy	1631 (65.1)	186 (56.5)	**0.003**	1964 (55.5)	2325 (61.8)	**<0.001**
AD RT	697 (27.8)	74 (22.5)	**0.042**	634 (17.9)	1004 (26.7)	**<0.001**
Operation			0.311			0.223
LP	432 (17.2)	48 (14.6)		627 (17.7)	665 (17.7)	
PD	1526 (60.9)	207 (62.9)		2185 (61.7)	2246 (59.7)	
TP	351 (14.0)	47 (14.3)		450 (12.7)	530 (14.1)	
Extended PD	133 (5.3)	16 (4.9)		178 (5.0)	199 (5.3)	
Pancreatectomy NOS	28 (1.1)	8 (2.4)		50 (1.4)	49 (1.3)	
Excision	12 (0.5)	0 (0.0)		16 (0.5)	20 (0.5)	
Unknown	25 (1.0)	3 (0.9)		34 (1.0)	55 (1.5)	
Pathologic T			0.571			**<0.001**
Tis	34 (1.4)	2 (0.6)		57 (1.6)	25 (0.7)	
T0	20 (0.8)	3 (0.9)		32 (0.9)	22 (0.6)	
T1	285 (11.4)	41 (12.5)		449 (12.7)	354 (9.4)	
T2	451 (18.0)	62 (18.8)		593 (16.8)	692 (18.4)	
T3	1573 (62.7)	199 (60.5)		2236 (63.2)	2415 (64.2)	
T4	37 (1.5)	2 (0.6)		40 (1.1)	85 (2.3)	
Tx	41 (1.6)	8 (2.4)		55 (1.6)	70 (1.9)	
Missing	66 (2.6)	12 (3.6)		78 (2.2)	101 (2.7)	
Pathologic N			0.630			**<0.001**
N0	1091 (43.5)	143 (43.5)		1635 (46.2)	1543 (41.0)	
N1	1299 (51.8)	166 (50.5)		1760 (49.7)	2028 (53.9)	
Nx	49 (2.0)	7 (2.1)		63 (1.8)	83 (2.2)	
Missing	68 (2.7)	13 (4.0)		82 (2.3)	110 (2.9)	
No. of nodes retrieved	17.1 ± 10.8	16.3 ± 10.0	0.185	17.4 ± 10.3	15.1 ± 9.8	**<0.001**
No. of nodes positive	2.20 ± 3.73	2.17 ± 3.58	0.920	1.90 ± 2.96	2.09 ± 3.36	**0.016**
Lymph node ratio	0.13 ± 0.18	0.13 ± 0.19	0.983	0.11 ± 0.17	0.14 ± 0.19	**<0.001**
Margins			0.396			**<0.001**
R0	2009 (80.1)	252 (76.6)		2913 (82.3)	2860 (76.0)	
R1	393 (15.7)	63 (19.1)		509 (14.4)	722 (19.2)	
R2	13 (0.5)	1 (0.3)		19 (0.5)	21 (0.6)	
Missing	92 (3.7)	13 (4.0)		99 (2.8)	161 (4.3)	
Length of stay, days	9.6 ± 8.6	10.5 ± 8.7	0.115	10.0 ± 8.7	10.7 ± 9.9	**0.002**
30-day readmission	208 (8.5)	18 (5.6)	0.074	300 (8.6)	312 (8.6)	0.962
30-day mortality	29 (1.4)	10 (3.7)	**0.007**	70 (2.4)	106 (3.5)	**0.012**
90-day mortality	54 (2.7)	16 (5.9)	**0.004**	139 (4.8)	211 (7.1)	**<0.001**

Bold numbers are for statistical significance (*p* < 0.05).

LP, left pancreatectomy; NAD, neoadjuvant; PD, pancreaticoduodenectomy; RT, radiotherapy; TP, total pancreatectomy.

## Discussion

Surgical resection is utilized in a minority of patients with early stage PDAC despite improvements in OS. This study demonstrates improved OS in patients who underwent operative intervention compared with those who did not undergo surgery or receive any treatment. There was a trend toward decreased utilization of operative intervention over the course of the study period. Sociodemographic factors predicting nonoperative management included older age, black or Hispanic race, care at community facilities, nonprivate health insurance, less education, and lower income. In patients who underwent operative intervention, the sociodemographic factors associated with lower OS included care at nonacademic facilities and Medicaid insurance. To our knowledge, this is the first study to describe these findings within the era of modern multidisciplinary and multimodality care of PDAC.

Our first objective was to reassess the utilization of operative intervention in the modern era of management of early stage PDAC. The landmark study by Bilimoria et al. in 2007 analyzed the NCDB between 1995 and 2004 and reported that 28.6% of patients with clinical stage I disease underwent pancreatectomy, whereas 54.7% of patients were never offered surgery.^3^ More recently, Fergus et al. demonstrated slightly improved utilization but overall similar findings by using data from the NCDB between 2004 and 2014.^20^ Both studies demonstrated, identified, and improved utilization of surgery over time. However, the majority of patients in both of these studies were treated before the current era, in which utilization of multiagent neoadjuvant therapy is increasing,^4,10–12^ there is more emphasis on prehabilitation,^13^ there is wider acceptance of minimally invasive pancreatectomy,^14–18^ and more effective adjuvant regimens have been described.^21^

In our study, we report that 41.2% of patients underwent operative intervention, but there was utilization of operative interventions over the study period from 46.8% to 38.9%. This decrease in operative intervention may reflect an increased utilization of neoadjuvant chemotherapy, with some patients failing to make it to operative resection. In fact, the gradual 8% decrease in surgical intervention over the study period is counterbalanced by an ∼8% increase in nonoperative management (Fig. 1A). Of the patients who underwent nonoperative management, 45.6% had no documented contraindication. The majority of patients who did not undergo operative intervention received no treatment whatsoever. Patients who underwent surgery had improved OS (21.5 months) compared with those who received nonoperative management (11.1 months) or no treatment (5.1 months), and lack of operative intervention was an independent predictor of mortality.

Our second objective was to identify variables that were associated with nonoperative management in early stage PDAC. Multiple studies report disparities in the treatment based on race and insurance status.^2,3,6–9^ For example, the studies by Bilimoria et al.^3^ and Fergus et al.^20^ also identify that age, race, income, education level, insurance status, and facility type were associated with choice of operative versus nonoperative management. We report similar results in our study, with age, black or Hispanic race, care at community facilities, nonprivate health insurance, lower education, lower median household income, cT2 tumors, pancreatic head tumors, and CA19-9 levels ≥38 U/mL all being independently associated with a decision for nonoperative management. These findings highlight that patient conditional, biological, and anatomical factors can influence treatment decisions, whereas sociodemographic factors continue to be associated with detrimental treatment decisions.

Our third objective was to analyze interactions between the sociodemographic risk factors that predicted nonoperative management and survival in patients who underwent surgery to understand whether these factors were associated with OS or were simply barriers to standard-of-care treatment of PDAC. After controlling for baseline characteristics, we demonstrate that patients with Medicare or Medicaid insurance, and those treated at nonacademic facilities, had worse OS after surgical intervention. Using the SEER database (2004–2011), Shapiro et al. demonstrate that sociodemographic variables that predicted nonoperative management in early PDAC did not impact survival in patients who underwent surgery, with the exception of the geographic region in the Southeast.^2^ However, the analysis in this study was limited by the inability to distinguish between insurance type (only insured vs. uninsured), and there are no data on facility type, education, or median income. Our results had some similarities, as race did not impact survival in patients who underwent surgery, but we were able to explore more deeply various sociodemographic variables associated with both treatment and survival. Prior studies using the NCDB^3,20^ have not explored the association between sociodemographic factors associated with nonoperative management and survival in patients who were operatively managed.

When compared with patients with private insurance, patients with Medicaid had lower rates of adjuvant therapy and higher rates of 30- and 90-day postoperative mortality. These data are similar to those reported by Sanford et al. after an analysis of the NCDB (2004–2015), who report that insurance status was associated with receipt of adjuvant therapy after pancreatectomy.^7^ Patients without insurance (OR 0.61) or on Medicaid (OR 0.61) were less likely to receive adjuvant chemotherapy after resection. Analysis of postoperative outcomes was not included in their study. Swanson et al. analyzed 30- and 90-day mortality after pancreatectomy by using the NCDB (2007–2010).^22^ The overall unadjusted 30- and 90-day mortality rates were 3.7% and 7.4%, respectively, and patients who lacked insurance or were on Medicaid had higher unadjusted mortality rates than those with private insurance. However, after risk adjustment, insurance type was not associated with postoperative mortality. Our data are in agreement with these findings. It is known that adjuvant therapy is associated with improved OS after pancreatectomy for PDAC,^21,23,24^ and if patients with Medicaid receive adjuvant therapy less frequently, shortened OS is to be expected. The reasons for increased 30- and 90-day postoperative mortality are less clear, but they may suggest barriers to care, delayed presentation, and inability to salvage patients who suffer postpancreatectomy complications.

When operative outcomes were compared based on facility type, patients treated at community facilities or integrated networks received lower rates of neoadjuvant therapy, had higher rates of nodal involvement and higher lymph node ratios, higher margin-positive resection rates, and higher 30- and 90-day postoperative mortality. Neoadjuvant therapy is associated with improved OS in early stage PDAC as well as lower rates of nodal involvement and higher rates of margin-negative resection,^25^ and it may contribute to the observed differences in pathologic outcomes at nonacademic facilities as neoadjuvant therapy was used less frequently in nonacademic settings (17.3% vs. 13.6%, Table 4). Chu et al. studied outcomes for stage I–III PDAC by using the NCDB (1998–2011) and reported that nonacademic facilities had lower volume of cases, higher rates of positive margins, and higher 30- and 90-day mortality,^26^ although with mortality rates much higher than those reported in our study. More recently, Sweigert et al. reported on the achievement of textbook outcomes (a composite definition that includes margin-negative resection, compliant lymph node evaluation, no prolonged length of stay, no 30-day readmission or mortality, and receipt of adjuvant chemotherapy; all criteria must be met) after pancreaticoduodenectomy for PDAC by using the NCDB from 2006 to 2016.^27^ The overall rate of textbook outcome was 16.8%, with higher rates at academic/research facilities compared with nonacademic centers (19.2% vs. 12.6%). Our data are concordant with these findings and suggest that overall, higher quality care is provided at academic/research centers that specialize in the multidisciplinary management of PDAC.

There are several potential limitations to this study. As with any large, national database there is an inherent risk of unknown confounders, as well as incorrect or missing data entry by staff at participating institutions. For example, the NCDB captures care at CoC facilities, raising the possibility that patients who ultimately underwent operative intervention at non-CoC facilities after extended neoadjuvant regimens were misclassified as receiving nonoperative management, which would affect our analyses on survival as well as risk factors for nonoperative management. This is of particular relevance since the utilization of neoadjuvant therapy increased over the study period (from 9.9% to 24.0% [data not shown]) concomitant with a decreasing trend of operative intervention over the same period. In addition, patients may have had disease progression on neoadjuvant therapy with intent for operative intervention, data not captured by the NCDB, and could result in a misclassified nonoperative treatment category. The use of AJCC clinical staging was used as a surrogate for early stage (cT1–T2) disease, as the NCDB does not contain assessment of resectability by consensus guidelines.^28,29^ The majority of patients who underwent resection had pT3 tumors (Table 4 and not shown), implying that multiple patients had anatomically borderline or locally advanced disease and thus may not have been offered surgery based on clinician judgement. In addition, patients with elevated CA19-9 levels and biologically borderline/high-risk disease may not have been offered surgery based on clinician judgement. Neither of these scenarios would be captured accurately with the inherent limitations of the NCDB. Further, the CA19-9 values captured within the NCDB have a cutoff of 98 U/mL and are absent in a known serum bilirubin concentration, making interpretation of CA19-9 data difficult.

## Conclusion

To our knowledge, this is the first study to report on the utilization of operative management of early stage pancreatic cancer by using NCDB data from the modern era of multidisciplinary and multimodality management of the disease process. We demonstrate that a minority of patients undergo surgery for early stage disease, and these patients have the longest OS. Analysis of nonclinical factors reveals persistent racial and socioeconomic barriers to receiving operative management, and a significant number of patients receive no treatment whatsoever. Further, the majority of the sociodemographic factors associated with nonoperative management were not associated with OS in patients who underwent surgery. However, patients with Medicaid and those who received care at nonacademic centers had shorter OS. The reasons for these findings are multifactorial and provide targets for future study and improvement in both access and quality of care.

## Abbreviations Used

AJCC: American Joint Committee on Cancer
CA19-9: carbohydrate antigen 19-9
CI: confidence interval
CoC: Commission on Cancer
HR: hazard ratio
HSD: high school diploma
ICD-O: *International Classification of Diseases for Oncology*
IQR: interquartile range
LP: left pancreatectomy
NAD: neoadjuvant
NCDB: National Cancer Database
OR: odds ratio
OS: overall survival
PD: pancreaticoduodenectomy
PDAC: pancreatic adenocarcinoma
RT: radiotherapy
SD: standard deviation
SEER: Surveillance, Epidemiology, and End Results
TP: total pancreatectomy
